# Data Quality Assessment of Gravity Recovery and Climate Experiment Follow-On Accelerometer

**DOI:** 10.3390/s24134286

**Published:** 2024-07-01

**Authors:** Zongpeng Pan, Yun Xiao

**Affiliations:** 1 Xi’an Research Institute of Surveying and Mapping, Xi’an 710054, China; 2 State Key Laboratory of Geo-Information Engineering, Xi’an 710054, China; 3 Key Laboratory of Smart Earth, Beijing 100029, China

**Keywords:** gravity satellite, gravity recovery and climate experiment follow-on, accelerometer, data quality assessment

## Abstract

Accelerometers are mainly used to measure the non-conservative forces at the center of mass of gravity satellites and are the core payloads of gravity satellites. All kinds of disturbances in the satellite platform and the environment will affect the quality of the accelerometer data. This paper focuses on the quality assessment of accelerometer data from the GRACE-FO satellites. Based on the ACC1A data, we focus on the analysis of accelerometer data anomalies caused by various types of disturbances in the satellite platform and environment, including thruster spikes, peaks, twangs, and magnetic torque disturbances. The data characteristics and data accuracy of the accelerometer in different operational states and satellite observation modes are analyzed using accelerometer observation data from different time periods. Finally, the data consistency of the accelerometer is analyzed using the accelerometer transplantation method. The results show that the amplitude spectral density of three-axis linear acceleration is better than the specified accuracy (above 10^−1^ Hz) in the accelerometer’s nominal status. The results are helpful for understanding the characteristics and data accuracy of GRACE-FO accelerometer observations.

## 1. Introduction

The GRACE-FO (Gravity Recovery and Climate Experiment Follow-On) mission was started in May 2018. The primary scientific mission of GRACE-FO is to accurately determine Earth’s static and time-variable gravity fields. GRACE-FO consists of two LEO satellites in the same near-circular, near-polar orbital plane, with the satellites about 220 km apart. The initial orbital altitude is about 500 km. The satellites carry core payloads such as a K-band ranging system (KBR), accelerometers (ACC), and GNSS receivers. In addition, GRACE-FO carries a laser range interferometer (LRI) to validate the contribution of inter-satellite laser ranging technology to future gravity mapping missions [1].

In the satellite gravity detection system, the satellite orbit is affected not only by conservative forces but also by non-conservative forces such as atmospheric drag, solar radiation pressure (SRP), and Earth radiation pressure (ERP). Accurate measurement of the non-conservative forces is the key to accurate orbit determination and high-precision gravity field recovery. The GRACE/GRACE-FO satellites are equipped with three-axis electrostatic accelerometers with a measurement accuracy of up to 10^−10^ m/s^2^, which are mainly used to observe the non-conservative forces at the center of mass of the satellite [2,3,4]. Due to the high sensitivity of the ACCs [5], different types of disturbances of the satellite platform, mainly temperature control switches, cold-gas thrusters thrust deviations, and magnetic torque disturbances, can affect the ACC data and introduce different types of anomalous signals [6,7,8,9,10,11].

Frommknecht et al. (2007) [12] analyzed different types of anomalies in the GRACE accelerometer data. The twang signal in the accelerometer data, with a magnitude of 10^−5^ m/s^2^ to 10^−7^ m/s^2^ and a duration of about 5 s, may be caused by the thermal effects of the thermal insulation at the base of the satellite. The peak signal in the acceleration data, which affects all three axes of the accelerometers by a comparable order of magnitude of up to 10^−8^ m/s^2^, is mainly caused by heater switches [3]. The operation of the magnetic torquer on the satellite also introduces anomalous signals in the accelerometer [13]. These anomalous signals are mainly concentrated in the high-frequency range above 0.1 Hz, and the amplitude spectral density in the low frequencies is lower than the accelerometer error model. In addition, the accelerometer data contain anomalous spikes caused by the thrust deviations of the attitude control thrusters, but these deviations are not treated in the ACC data pre-processing [3].

With the launch of the GRACE-FO satellite, the SDS (GRACE-FO Science Data System) released the raw Level-1A accelerometer data (ACC1A, Accelerometer Level-1A Data) of the GRACE-FO in 2019, as well as the calibrated ACT1A data (Calibrated Accelerometer Level-1A Data). Anomalies due to thruster thrust bias were corrected in the ACT1A data, and an ACT1B data product was generated based on this product [10]. Since 21 June 2018, an anomaly jump has been observed in the GRACE-D accelerometer data during the GRACE-D thruster operation. The GRACE-D accelerometer data degraded. The current data processing strategy for the GRACE-D accelerometer is to use GRACE-C transplant accelerometer data instead [11,14]. Pre-processing methods for raw GRACE-D accelerometer data are still under investigation.

The assessment of the data quality of the GRACE/GRACE-FO accelerometer is a complex task. As the sensitivity and accuracy of the ACC sensors are unprecedented in an LEO environment, validation is inherently difficult. Meanwhile, the measurement noise of the accelerometers and the environmental disturbances of the satellites overlap and cannot be separated, so there are many challenges in directly assessing the quality and accuracy of the accelerometer data. The quality of accelerometer data is typically evaluated indirectly based on the accuracy of the gravity field model or the accuracy of the satellite’s orbit [15,16,17]. A direct way to quantify the accuracy of accelerometer data is to analyze the high-frequency observation noise based on raw 10 Hz observations [2,12,18]. However, the different types of spikes in accelerometer data limit the generality of this approach, and ‘clean’ data segments free of spikes must be selected for analysis. Another method of validating the quality of accelerometer data is to compare the transplant of GRACE-FO ACC data. Since the GRACE-FO satellites follow each other in a similar orbital plane, with the satellites about 220 km apart, the tracking satellite will pass through similar spatial positions and environments of the lead satellite in a short time (about 30 s), which are subject to essentially the same non-conservative forces. The difference in transplant ACC data can be analyzed in terms of the measurement noise characteristics of the accelerometers [9,19]. Transplant ACC data can also be used to recover missing data when a satellite’s accelerometers are operating abnormally.

This paper investigates the characteristics of the accelerometer data from the GRACE-FO satellites and assesses the quality of the accelerometer data at different times. The magnitude and characteristics of anomalies in the accelerometer data, such as thrust deviation of the attitude control thruster, magnetic torquer interference, temperature control switching effect, and twangs, are analyzed. The data characteristics and data accuracy of accelerometers in different operational states and satellite observation modes are analyzed. Finally, the data consistency of the GRACE-FO ACC data is analyzed using the transplant method. The rest of the paper is organized as follows: the second part is the accelerometer data processing method; the third part is the experimental strategy and the analysis of the experimental results; and finally, the conclusion of this paper is given.

## 2. Accelerometer Data Processing Methods

### 2.1. Accelerometer Data and On-Orbit Status

The GRACE-FO accelerometer is a three-axis electrostatic accelerometer that is mainly used to observe the non-conservative forces at the satellite’s center of mass, including atmospheric drag, SRP, ERP, and other perturbations. The design specifications of the accelerometer are shown in Table 1. 

There are three types of scientific data products for the GRACE-FO accelerometer data. The ACC1A data products contain the 10-Hz linear acceleration measurements given in the accelerometer reference frame (AF). The time frame of the data is determined by the on-board computer (OBC). To obtain better gravity field solutions, a series of calibration processes are applied to ACC1A, providing the calibrated accelerometer data products (ACT1A) [10]. During Level-1A to Level-1B data processing, the ACT1A linear accelerations are processed, time-tagged, and low-pass filtered to reduce high-frequency measurement noise [20]. The output of this process is a 1 Hz ACT1B product, given in Satellite Reference Frame (SRF) and GPS time. 

The GRACE-FO accelerometer operates in two main modes: Normal Range Mode (NRM) and Large Range Mode (LRM). LRM provides better control of the test mass at the expense of lower acceleration resolution, while NRM is designed for nominal science operations and provides the best acceleration resolution at the expense of less robust control of the test mass. After the launch of the satellites on 22 May 2018, the accelerometers of the GRACE-C/D operated normally until 21 June 2018. After that, the GRACE-D accelerometer data showed anomalies, and there were jumps in the accelerometer observations during GRACE-D thruster firing. To reduce the frequency of thruster activation, the attitude control mode of GRACE-FO was changed from nominal AOCS mode (with pointing offsets up to +/− 1 deg) to relaxed pointing mode in January–February 2023. The relaxed pointing mode is intended to reduce thruster activation and overall fuel consumption. The GRACE-C/D are collecting science data in a relaxed AOCS pointing mode, with pointing offsets up to +/− 2 deg.

### 2.2. Accelerometer Data Anomaly Signal Extraction

Due to the accuracy and sensitivity of the accelerometers, various types of perturbations of the satellite platform can affect the linear acceleration and introduce various types of anomalous signals. Therefore, the accelerometer observations contain not only non-conservative forces such as atmospheric drag, SRP, and ERP but also various types of anomalous signals. In order to analyze the effects of the various types of anomalies (mainly distributed in the high-frequency part), the accelerometer data can be high-pass filtered to remove the low-frequency signals and then analyze the characteristics of the various types of anomalies. In this paper, the following processing strategy is adopted: firstly, the various types of anomalous signals in the accelerometer data are removed, then the interpolation is conducted to make up the data gaps, the accelerometer measurement signal is extracted by low-pass filtering, and finally, the original ACC data are deducted from the low-pass filtered accelerometer measurement signal to obtain the high-pass filtering data.

The empirical threshold method is used to detect and process anomalous signals. As non-conservative forces change slowly in a short period of time, they can construct anomalous detection values and the empirical threshold for comparison (as shown in Equation (1)). If there is a sudden jump point that exceeds the empirical threshold and is judged to be anomalous, the anomalous data are rejected and interpolated to make up the data gaps (data interpolation can be used as a linear interpolation or a low-order polynomial interpolation method).

(1) $$\Delta a=a_{k}-\frac{1}{n-1}\sum_{k-n}^{k-1}a_{i}$$

where Δa is the anomalous detection value, $a_{k}$ is the current ACC data, $a_{i}$ is the ACC data before k.

The empirical thresholds for outlier removal of the GRACE-FO accelerometer data are shown in Table 2 [10]. Since the orbital altitude of the GRACE-FO is around 300 km to 500 km and the non-conservative force variations in the space environment are less than 10^−7^ m/s^2^, the outlier thresholds are mainly set based on this. For the outlier less than the threshold, it is not processed at present, but the influence of the outlier is suppressed by low-pass filtering.

For the spike anomalies caused by the cold-gas thruster firings, we mainly identify the time period of the thrust anomalies in the three-axis acceleration data based on the THR1A data. Removing a time span of one second before and after the spikes caused by thruster firings and filling the gaps with linear interpolation.

The CRN digital filter can be used to eliminate or suppress the high-frequency noise in ACC1A data. The CRN filter [20] has been used for data filtering of all kinds of payloads on the GRACE satellite. In this paper, we refer to the GRACE mission that uses the CRN filter and its parameter settings to low-pass filter the accelerometer data of the GRACE-FO.

The CRN filter can be expressed by the following equation [20]:

(2) $$a_{i}^{out}=\sum_{n=-N_{h}}^{N_{h}}F_{n}a_{i-n}^{raw}$$

where $F_{n}$ is the time-domain filter weight function, $a_{i-n}^{raw}$ is the input data, $a_{i}^{out}$ is the output data, and *N_h_* = (*N_f_* − 1)/2. The specific form of the filter weight function and filter parameters can be found in the literature [20].

### 2.3. Accelerometer Data Transplant Methodology

On 21 June 2018, the GRACE-FO GRACE-D accelerometer data was anomalous, with jumps in accelerometer observations caused by GRACE-D thrusters firing. The current data processing strategy for GRACE-D accelerometers is to use GRACE-C transplanted accelerometer data instead. McCullough et al. (2019) [10] provide detailed accelerometer data transplant methods and steps. In this paper, the accelerometer data transplant method is mainly used for comparative analysis of the data quality of GRACE-C/D accelerometers under normal operating conditions. The data transplant process is slightly different, which is mainly to eliminate anomalous data, such as thruster spikes in accelerometer data, and then transplant them for comparison.

Assuming that the GRACE-C accelerometer data are transplanted to GRACE-D, the anomalies and thruster firing events in the GRACE-C accelerometer data (before and after 1 s of data) are removed and interpolated to fill the data gaps. Similar operations are conducted for GRACE-D. 

Then, using the precision orbits of both satellites, the transplant time correction of the GRACE-C ACC data is calculated and corrected to the observation time tags. The attitude correction of the transplant linear acceleration data is performed by using the attitude data of the star cameras. Finally, the GRACE-C accelerometer data are transplanted to GRACE-D and then compared to the GRACE-D accelerometer observations. Before the comparison, de-biasing and de-drift correction of the ACC data are also required to ensure the consistency of the data.

## 3. Experiment and Result Analysis

Based on the GRACE-FO ACC1A data, the data processing strategy in Section 2 is adopted to focus on analyzing the accelerometer data anomalies caused by various types of disturbances in the satellite platform and the environment. Through the accelerometer observation data in different time periods, we analyze the data characteristics and data accuracy of the accelerometer in different operational status, and satellite observation modes. The observation time of the ACC1A, the status of the GRACE-C/D accelerometer, and the attitude control mode of the satellites are shown in Table 3.

Accelerometer data analysis also uses data products such as THR1A, GNI1B, CLK1B, and TIM1B. THR1A data are thruster event data used to mark the moment of occurrence of thruster firing; GNI1B are precision orbit products used for satellite orbital position and velocity calculations; and TIM1B and CLK1B are clock error products used for accelerometer observations to correct the time tags. 

### 3.1. Analysis of Various Types of Anomalies in ACC1A Data

**Figure 1:**
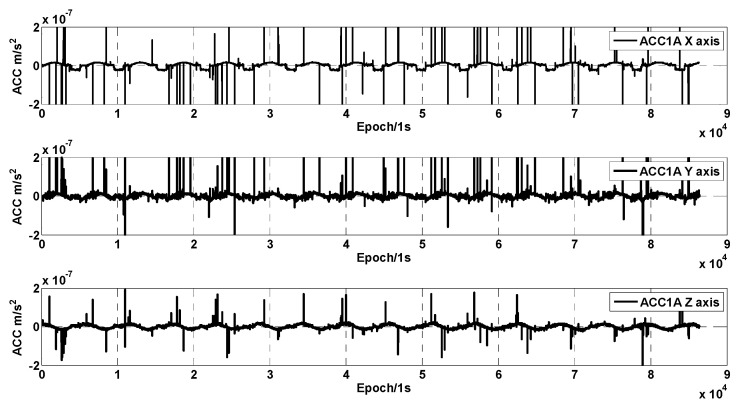
Linear acceleration of GRACE-C ACC1A data.

The GRACE-C accelerometer ACC1A data from 1 January 2019 was used to analyze the magnitude of various types of anomalous effects, such as thrust deviation of the attitude control thruster, magnetic torquer interference, temperature control switch effects, and twangs. Figure 1 shows the ACC1A data for three-axis linear acceleration. It can be seen that there are a large number of anomalous signals in the three-axis linear acceleration. The size of the non-conservative force in the orbit of the GRACE-FO satellite is in the order of 10^−8^ m/s^2^. However, due to various on-board disturbances, especially the work of the cold-gas thrusters, which introduce many anomalous signals, the non-conservative force measured by the accelerometers is masked by various anomalies.

**Figure 2:**
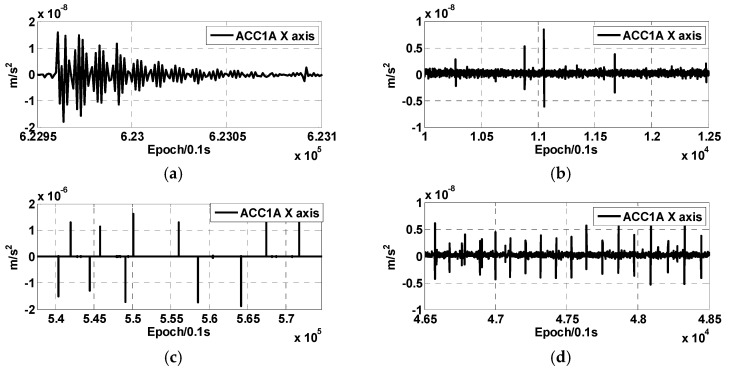
(**a**) Twangs disturbance; (**b**) peaks caused by switching of the heating system; (**c**) spikes caused by deviation of the thruster; and (**d**) peaks caused by magnetic torquer device operation in GRACE-C ACC1A data.

To better analyze the effects of various types of anomalies in ACC data, CRN high-pass filtering was performed on ACC1A to remove low-frequency signals (various types of anomalies are mainly high-frequency signals). Figure 2 shows the twang disturbance, peaks caused by switching of the heating system, spikes caused by the deviation of the thruster, and peaks caused by magnetic torquer device operation in GRACE-C ACC1A X-axis data. As can be seen in the figure, similar to the GRACE accelerometer data, the GRACE-FO accelerometer data also have damping oscillation anomalies twang signals, but their magnitude is smaller compared to the GRACE accelerometer data at about 10^−8^ m/s^2^ [12], with a duration of about 5 s. The peak signal is introduced by the temperature control switch, with a magnitude of 10^−8^ m/s^2^. Due to the frequent activation of the temperature control switch and its irregularity, there are a large number of peaks in the ACC data. The peak signal introduced by the operation of the magnetic torquer is related to the operation mechanism of the magnetic torquer, with a magnitude of 10^−8^ m/s^2^ and the characteristic of temporal distribution. The spike signal introduced by the thrust deviation of the cold-gas thrusters is recorded in the THR1A file, and their effects on the different axes of the accelerometer are of different magnitudes, with a maximum magnitude of 10^−5^ to 10^−6^ m/s^2^, and their duration is related to the time of the thruster firing.

### 3.2. Data Analysis of Accelerometer Normal Operating Status

**Figure 3:**
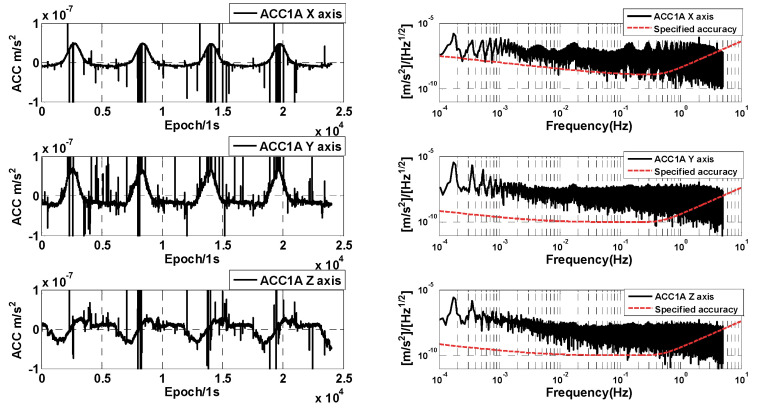
Time series and amplitude spectral density of GRACE-C ACC1A data.

**Figure 4:**
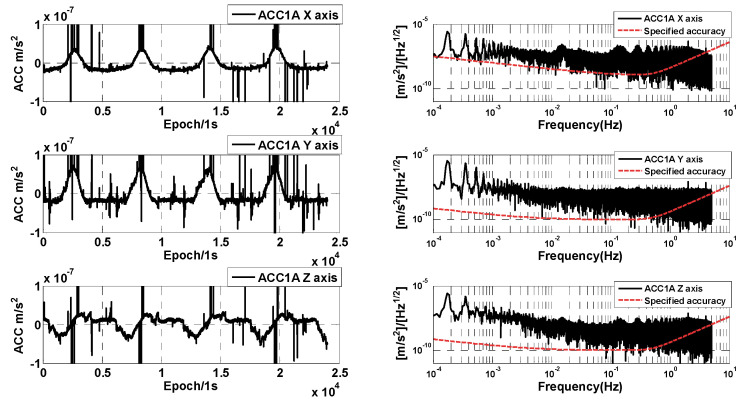
Time series and amplitude spectral densities of GRACE-D ACC1A data.

The ACC1A data of the GRACE-C/D are compared and analyzed under the normal operating conditions of the GRACE-C/D accelerometers. The raw data time series and amplitude spectral densities are first compared, and the ACC1A data of the GRACE-C/D on 1 June 2018 are shown in Figure 3 and Figure 4, where the time series and amplitude spectral density of the three-axis linear acceleration are presented. In this case, the time series are given only for a part of the time period (about four orbital cycles).

From Figure 3 and Figure 4, it can be seen that the three-axis non-conservative forces measured by the GRACE-C accelerometer are similar to the GRACE-D ACC data. However, there is a difference between the moment of thrust occurrence and the moment of magnetic torquer disturbance occurrence, which is related to the different attitude control strategies of the GRACE-C/D. The magnitude of the amplitude spectral densities of GRACE-C ACC data is also similar to the amplitude spectral densities of GRACE-D ACC data. The amplitude spectral densities of the one-day data segment are above the specified accuracy, which is mainly due to the effect of the superposition of the non-conservative force signals and other kinds of anomalies. 

**Figure 5:**
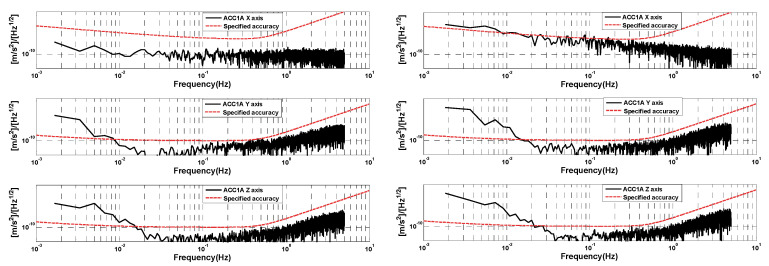
Amplitude spectral densities of selected data segments of GRACE-C (**left**) and GRACE-D (**right**) ACC1A.

In order to better analyze the measurement accuracy of the GRACE-C/D accelerometers, we selected the data segments that are free from thruster firing (about 600 s of data) to analyze and compare the amplitude spectral density. Figure 5 shows the amplitude spectral density of the selected data segments of GRACE-C and GRACE-D ACC1A. It can be seen that the amplitude spectral density of three-axis linear acceleration is better than the specified accuracy (above 10^−1^ Hz). The performance of the high-sensitivity axis amplitude spectral density of GRACE-C ACC data is consistent with that of GRACE-D ACC data. However, the amplitude spectral density of the low-sensitivity axis of GRACE-D ACC data is obviously larger than that of GRACE-C ACC data.

**Figure 6:**
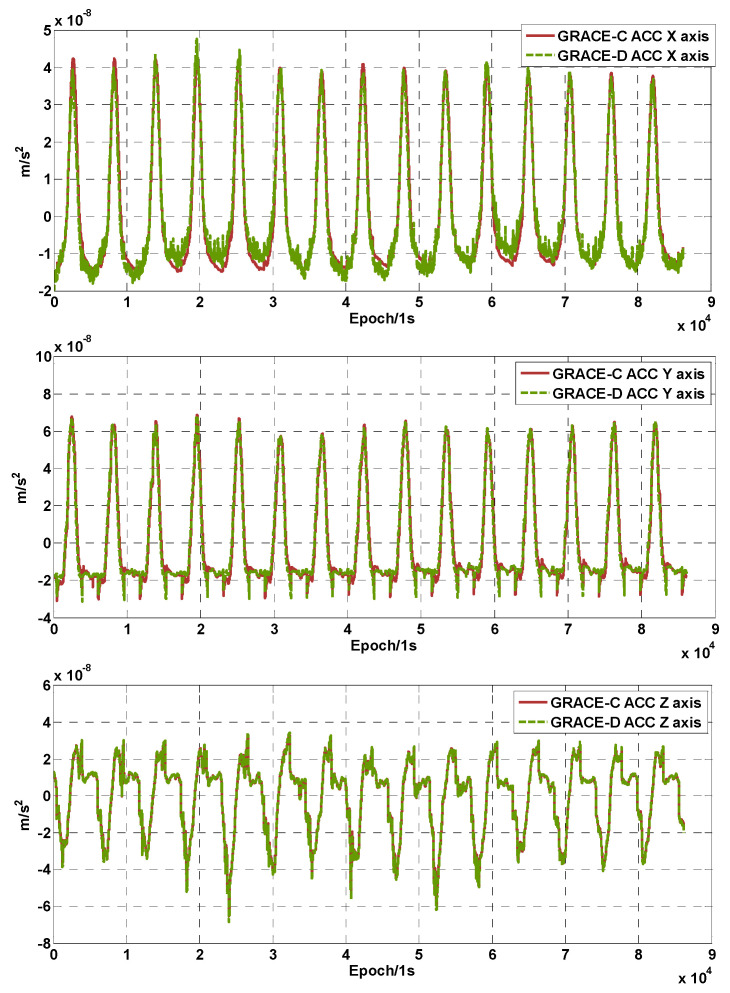
Three-axis linear acceleration of GRACE-C and GRACE-D.

To further analyze the cause of this phenomenon, the GRACE-C and GRACE-D accelerometer data were transplanted and compared using the method described in Section 2. Figure 6 shows the three-axis linear acceleration of the GRACE-C/D ACC after transplant. It can be seen that the non-conservative force consistency of the GRACE-C/D accelerometer measurements on the Y/Z axis (high-sensitivity axis) is very good. While in the low-sensitivity axis, the GRACE-D acceleration data have obvious perturbations related to the presence of scintillation noise in the X3 pole plate voltage [11].

### 3.3. Data Analysis of Accelerometer Abnormal Operating Status

**Figure 7:**
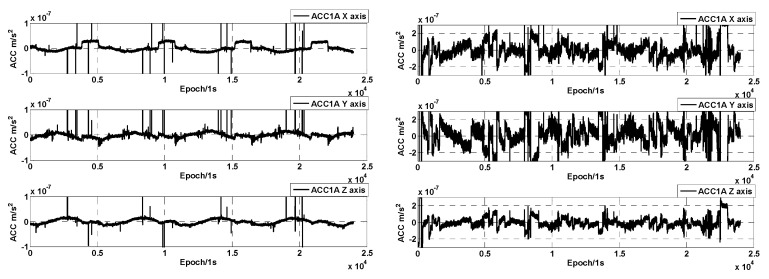
GRACE-D ACC linear acceleration on 1 January 2019 (**left**) and 8 June 2019 (**right**).

After 21 June 2018, GRACE-D accelerometer data showed anomalies, and there were jumps in the accelerometer observations during GRACE-D thrusters firing. The GRACE-D ACC1A data characteristics were analyzed in both small and large-range modes. The three-axis linear acceleration time series of GRACE-D ACC1A data on 1 January 2019 and 8 June 2019 are shown in Figure 7. On 1 January 2019, the GRACE-D accelerometer was in small range mode, and on 1 June 2019, the GRACE-D accelerometer was in large range mode. 

**Figure 8:**
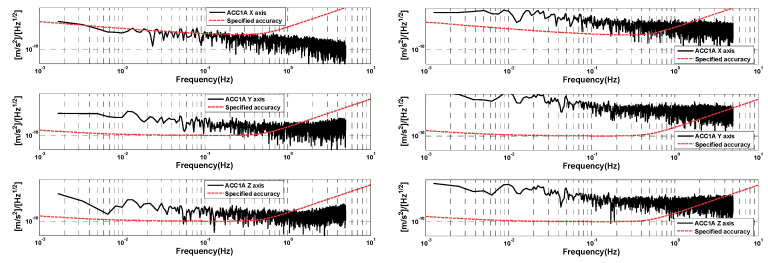
Amplitude spectral densities of selected data segments of GRACE-D ACC on 1 January 2019 (**left**) and 8 June 2019 (**right**).

As can be seen in Figure 7 (right), the noise of the ACC data becomes significantly bigger in the large range mode, and the jumps in the ACC data before and after the occurrence of the thrust are evident. However, in the small-range case on 1 January 2019, the jumps in the ACC data are smaller. The amplitude spectral density of selected data segments of GRACE-D ACC at small and large ranges is shown in Figure 8. Compared with Figure 5 (normal operating mode of the accelerometers), the amplitude spectral densities of the three-axis linear accelerations of the GRACE-D accelerometer are all significantly larger than those of the GRACE-C and are more pronounced in the large range mode.

### 3.4. Analysis of Accelerometer Data for Different Pointing Modes

**Figure 9:**
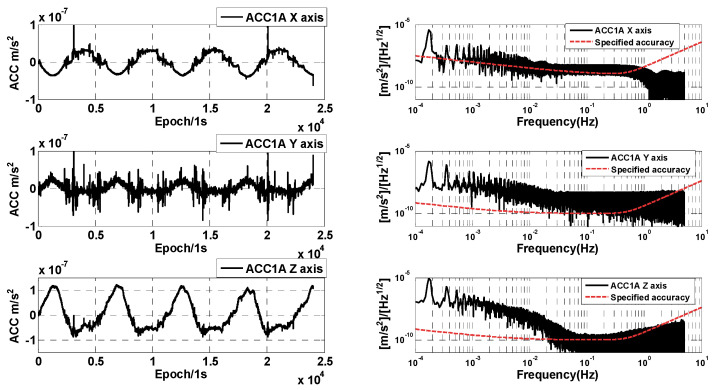
Time series (**left**) and amplitude spectral densities (**right**) of GRACE-C ACC1A data in relaxed inter-satellite pointing mode.

In order to save fuel in the cylinders and reduce thruster working time and frequency, the satellite attitude control mode was changed from the normal inter-satellite pointing mode to the relaxed inter-satellite pointing mode from January to February 2023. The thruster working frequency is obviously reduced in this mode, and the three-axis linear acceleration of the GRACE-C accelerometer on 3 February 2023 is shown in Figure 9. Comparing with Figure 3, it can be found that the disturbances in the three-axis linear acceleration are obviously reduced for the relaxed inter-satellite pointing mode compared to the normal inter-satellite pointing mode. This is mainly related to the attitude control strategy, and the frequency of thruster operation is reduced. Similar results are also found for the GRACE-D accelerometer, which will not be given again. Meanwhile, it can also be seen from the spectral analysis in Figure 9 that the overall amplitude spectral density of the single-day data is much smaller under the relaxed inter-satellite pointing mode, and in the high-frequency part (greater than 0.1 Hz), it is close to the specified accuracy.

**Figure 10:**
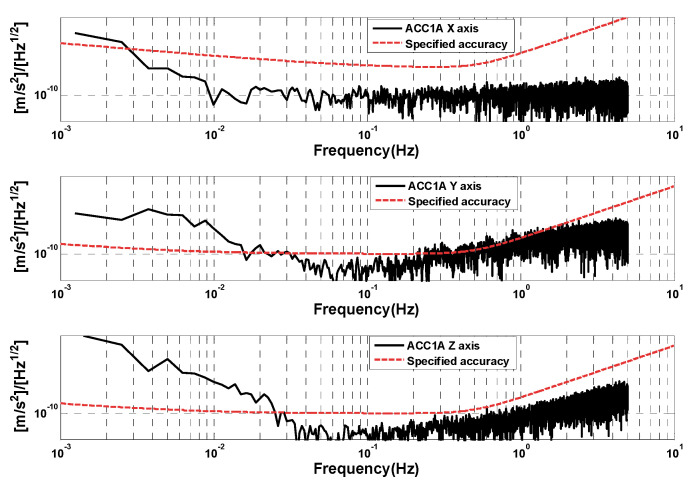
Amplitude spectral densities of selected data segments of GRACE-C ACC in relaxed pointing mode.

In order to further analyze the accuracy of the accelerometer measurements in the relaxed pointing mode, we select the no-thruster operation period (about 600 s of data) to analyze and compare the amplitude spectral density of the GRACE-C accelerometer. The amplitude spectral density of the GRACE-C accelerometer is given in Figure 10. It can be seen from the figure that for the relaxed pointing mode, the three-axis linear acceleration amplitude spectral density is basically the same as that of the normal inter-satellite pointing mode.

## 4. Conclusions

This paper focuses on the quality assessment of accelerometer data from the GRACE-FO satellites. Based on the ACC1A data, we focus on analyzing the magnitude of anomalies in the accelerometer data, such as thrust deviation of the attitude control thruster, magnetic torquer interference, temperature control switch influence, and twangs. The data characteristics and data accuracy of the accelerometer in different operational states and satellite attitude control modes are analyzed using accelerometer data in different time periods. The results show that:

(1) There are a large number of anomalous signals in the GRACE-FO ACC1A data. The magnitude of the twangs and peaks introduced by the temperature control switch and the operation of the magnetic torquer is about 10^−8^ m/s^2^. The spike signal is introduced by the thrust deviation of thrusters, with a maximum magnitude of 10^−5^ to 10^−6^ m/s^2^, and its duration is related to the time of the thruster firing.
(2) In the normal operating status of the accelerometer, the non-conservative forces measured by the GRACE-C accelerometer are similar to the GRACE-D ACC data. The amplitude spectral density of three-axis linear acceleration is better than the specified accuracy (above 10^−1^ Hz). The performance of the high-sensitivity axis amplitude spectral density of GRACE-C ACC data is consistent with that of GRACE-D ACC data. However, the amplitude spectral density of the low-sensitivity axis of GRACE-D ACC data is obviously larger than that of GRACE-C ACC data.
(3) After 21 June 2018, GRACE-D accelerometer data showed anomalies, and there were jumps in the accelerometer observations during GRACE-D thrusters firing. The amplitude spectral densities of the three-axis linear accelerations of the GRACE-D accelerometer are all significantly larger than those of the GRACE-C and are more pronounced in the large-range mode.
(4) Compared with the normal inter-satellite pointing mode, the disturbances in the three-axis linear acceleration are obviously reduced in the relaxed inter-satellite pointing mode. During the no-thruster operation period, the amplitude spectral density of the GRACE-C linear acceleration in the relaxed inter-satellite pointing mode is similar to that of linear acceleration in the normal inter-satellite pointing mode.

## Figures and Tables

**Table 1:** GRACE-FO linear acceleration requirements (in accelerometer reference frame) [1].

Linear Acceleration	X	Y/Z
Measurement range	5 × 10^−4^ m/s^2^	5 × 10^−5^ m/s^2^
Specified accuracy	10^−9^ × [1 + (f/0.5 Hz)^4^ + (0.1 Hz/f)]^1/2^ m/s^2^/(Hz)^1/2^	10^−10^ × [1 + (f/0.5 Hz)^4^ + (0.005 Hz/f)]^1/2^ m/s^2^/(Hz)^1/2^

**Table 2:** GRACE-FO acceleration threshold for outlier removal (in ARF).

	X	Y	Z
**Outlier threshold**	$\pm 1.0\times 10^{-7}m/s^{2}$	$\pm 3.0\times 10^{-7}m/s^{2}$	$\pm 1.5\times 10^{-7}m/s^{2}$

**Table 3:** Accelerometer status and satellite attitude control modes at different time periods.

Observation Time	GRACE-C ACC Status	GRACE-D ACC Status	Pointing Mode
1 June 2018	Nominal	Nominal	Nominal pointing mode
1 January 2019	Nominal	Anomalous	Nominal pointing mode
8 June 2019	Nominal	Anomalous (LRM)	Nominal pointing mode
3 February 2023	Nominal	Anomalous	Relaxed pointing mode

## Data Availability

The data in this article can be accessed on https://isdc.gfz-potsdam.de/grace-fo-isdc/grace-fo-gravity-data-and-documentation/?L=0 or the data can be available requesting from the corresponding author.
